# Antibacterial activity of bioactive anthraquinones isolated from *Cassia tora* L. against pathogenic intestinal microorganisms

**DOI:** 10.1007/s00210-026-05084-4

**Published:** 2026-02-13

**Authors:** Sri Renukadevi Balusamy, Seungah Lee, Gokulanathan Anandapadmanaban, Abdus Samad, Md Ripon Shikder, Chinnadurai Veeramani, Mohammed A. Alsaif, Khalid S. Al-Numair, Priyanka Singh, Haribalan Perumalsamy

**Affiliations:** 1 Department of Food Science and Biotechnology, Sejong University, Gwangjin-Gu, Seoul, Republic of Korea; 2 Department of Applied Chemistry and Institute of Natural Sciences, Kyung Hee University, Yongin-Si, Gyeonggi-Do 17104 Republic of Korea; 3 Plant Quarantine Technology Center, Animal and Plant Quarantine Agency, Gimcheon, 39660 Republic of Korea; 4 Graduate School of Biotechnology, College of Life Science, Kyung Hee University, Yongin-Si, 17104 Republic of Korea; 5 Department of Community Health Sciences, College of Applied Medical Sciences, King Saud University, Riyadh, P.O. Box 10219, 11433 Saudi Arabia; 6 The Novo Nordisk Foundation Center for Biosustainability, Technical University of Denmark, Kongens Lyngby, 2800 Denmark; 7 Center for Creative Convergence Education, Hanyang University, Seoul, 04763 Republic of Korea; 8 Research Institute for Convergence of Basic Science, Hanyang University, Seoul, 04763 Republic of Korea

**Keywords:** Antimicrobial resistance, *Cassia tora*, Anthraquinones, Intestinal pathogens, Natural antimicrobials, Antibacterial efficacy, Molecular docking

## Abstract

Antimicrobial resistance (AMR) poses a major global health challenge, particularly in intestinal infections where the efficacy of conventional antibiotics is rapidly declining. The rising prevalence of antibiotic-resistant pathogens underscores the urgent need for alternative antimicrobial agents with favorable activity profiles. This study evaluated the antibacterial potential of bioactive anthraquinones isolated from *Cassia tora* L. seeds against clinically relevant intestinal pathogens and probiotic bacteria. Five key anthraquinones, rhein, physcion, anthraquinone-2-carboxylic acid, emodin, and aloe-emodin, were identified and tested for their antibacterial activity. The anthraquinone metabolites exhibited strong antibacterial activity against pathogenic bacteria (*Escherichia coli*, *Staphylococcus aureus*, *Clostridium difficile*, etc.), while showing moderate to relatively lower inhibitory effects against beneficial probiotic strains such as *Clostridium butyricum*, *Bifidobacterium bifidum*, and *Lactobacillus casei*. The MIC profiles indicate relative or modest selectivity toward pathogens, rather than a complete sparing of probiotic species. Molecular docking analysis revealed that these metabolites bind favorably to key bacterial resistance proteins, often surpassing ciprofloxacin in binding affinity, supporting their observed antibacterial activity. Together, these results suggest that *C. tora* anthraquinones preferentially inhibit intestinal pathogens, maintaining a comparative margin of safety for beneficial gut flora. Anthraquinones isolated from *C. tora* seeds exhibit potent antibacterial activity toward pathogenic intestinal bacteria and relatively reduced activity against probiotic strains, reflecting selective preference in intestinal bacteria. These findings support their potential as natural antimicrobial candidates for managing intestinal infections and contributing to AMR mitigation. Nonetheless, the measurable inhibitory effects on probiotic species emphasize the need for careful dose optimization and microbiome-conscious therapeutic strategies. Further studies focusing on the mechanism of action, pharmacokinetics, toxicity, and in vivo efficacy are required to evaluate their clinical applicability.

**Supplementary Information:**

The online version contains supplementary material available at 10.1007/s00210-026-05084-4.

## Introduction

The gastrointestinal tract serves as a prominent reservoir for antibiotic-resistant organisms. In a healthy state, the gut microbiome constitutes a stable and diverse community that provides essential benefits to the host, including nutrient assimilation and protection against pathogenic microorganisms. However, the use of antibiotics can disrupt this delicate ecosystem by altering its taxonomic and functional composition, thereby facilitating the colonization and proliferation of pathogens (Sorbara et al. 2019; Anthony et al. 2021). The resilience of a healthy gut microbiome is attributed to its complex diversity, which inherently resists the establishment and growth of harmful pathogens (Kim et al. 2017).

AMR has emerged as one of the most pressing global health threats of the twenty-first century (Krishnaprasad & Kumar, 2024). According to the World Health Organization (WHO), antibiotic-resistant pathogens cause more than 700,000 deaths annually, a number projected to exceed 10 million by 2050 if new countermeasures are not developed. The gastrointestinal tract plays a dual role in this crisis: it acts both as a site of infection for multidrug-resistant enteric bacteria such as *Escherichia coli* and *Clostridium difficile*, and as a critical reservoir facilitating resistance gene exchange (Krishnaprasad & Kumar, 2024). The disruption of gut microbiota composition, commonly known as intestinal dysbiosis, has been linked to an increased risk of pathogen colonization and infection (Weiss & Hennet, 2017). This underscores the urgent need for novel antimicrobial strategies that selectively target pathogenic bacteria while preserving beneficial gut flora.

In May 2024, the WHO updated its Bacterial Priority Pathogens List (BPPL), highlighting several intestinal pathogens, including *E. coli*, *Salmonella* spp., *Clostridium difficile*, and *Staphylococcus aureus* as urgent or high-priority threats requiring new antimicrobial strategies. This update, along with the 2024 WHO List of Medically Important Antimicrobials (Jesudason 2024), underscores the global need for alternative agents with novel mechanisms of action and reduced risk of resistance development. These priorities directly motivate the exploration of plant-derived metabolites such as anthraquinones from *Cassia tora*, which have shown antimicrobial potential and may offer new avenues for targeting drug-resistant intestinal pathogens.

Plant-derived metabolites have recently gained attention as promising candidates for combating multidrug-resistant bacteria. Phytochemicals such as alkaloids, terpenoids, tannins, steroids, coumarins, and flavonoids exhibit potent antimicrobial and resistance-modulating properties. Their structural diversity enables multiple mechanisms of action, including interference with bacterial cell walls, efflux pump inhibition, and synergistic interactions with conventional antibiotics (Seukep et al., 2025). These advantages make plant metabolites particularly appealing for the development of safer, microbiota-friendly therapeutics.

Among such plants, *C. tora* L. (syn. Senna tora (L.) Roxb.), commonly known as sickle senna, is a member of the *Caesalpiniaceae* family widely cultivated across tropical Asia and recognized in traditional medicine systems for its pharmacological properties (Nawabjohn et al. 2022). In traditional Chinese and Ayurvedic practices, *C. tora* seeds are used for treating skin disorders, gastrointestinal ailments, and inflammatory diseases (Peng et al. 2022; Burbure et al., 2020). The seeds are rich in anthraquinone derivatives such as rhein, physcion, anthraquinone-2-carboxylic acid, emodin, and aloe-emodin, metabolites known for their diverse biological activities, including antioxidant, anti-inflammatory, and antimicrobial effects (Islam et al., 2018). Despite multiple reports of crude extract activity, the specific antibacterial profiles and selectivity of individual anthraquinones against both pathogenic and probiotic intestinal bacteria remain insufficiently characterized.

Recent studies have highlighted the antimicrobial potential of *C. tora*-based formulations. For instance, its alcoholic extract inhibited *Candida albicans*, while both alcoholic and aqueous extracts demonstrated broad antibacterial efficacy against intestinal pathogens. Moreover, *C. tora*-derived silver nanoparticles (AgNPs) showed pronounced antibacterial activity against *Staphylococcus aureus* (Nawabjohn et al. 2022). However, comparative data distinguishing effects on beneficial gut bacteria versus pathogens are lacking, an aspect crucial for the development of microbiota-safe antimicrobials.

Therefore, the present study aimed to isolate and characterize the major anthraquinone metabolites from *C. tora* and to evaluate their antibacterial activities against both pathogenic and beneficial intestinal microorganisms. By determining the minimum inhibitory concentrations (MICs) and assessing antibacterial activity, this work provides new insights into how *C. tora* anthraquinones may contribute to combating AMR while minimizing disruption of the gut microbiome.

## Materials and methods

### Instrumental analyses

^1^H and ^13^C NMR spectra were recorded in CDCl_3_ or MeOD on a Bruker AVANCE 600 spectrometer (Karlsruhe, Germany) using tetramethylsilane as an internal standard, and chemical shifts are given in δ (ppm). UV spectra were obtained in ethanol on a Kontron UVICON 933/934 spectrophotometer (Milan, Italy), FT-IR spectra on a Thermo Scientific Nicolet 6700 FT-IR (Madison, WI, USA), and mass spectra on a Jeol JMS-DX 303 spectrometer (Tokyo, Japan). Optical rotation was measured with a Rudolph Research Analytical Autopol III polarimeter (Flanders, NJ, USA). Merck silica gel (0.063–0.2 mm) (Darmstadt, Germany) was used for column chromatography. Merck precoated silica gel plates (Kieselgel 60 F_254_) were used for analytical thin-layer chromatography (TLC). Merck preparative silica gel plates and an Agilent 1200 series high-performance liquid chromatograph (HPLC) (Santa Clara, CA, USA) were used for the isolation of active principles.

### Bacterial strains and culture conditions

Five harmful intestinal bacteria, two non-pathogenic intestinal bacteria, six probiotic strains, and one acidulating bacterium examined in this study are listed in Table 1. They were purchased from the American Type Culture Collection (ATCC). Stock cultures of these bacterial strains were routinely stored in BHI broth (Becton, Dickinson and Company, Sparks, MD, USA) as reported previously (Tang et al. 2013), and, when required, were sub-cultured on BHI broth (pH 7.6). The cultures were incubated at 37 °C for 1 day in an atmosphere of 5% H_2_, 15% CO_2_, and 80% N_2_ in a Hirayama anaerobic chamber (Tokyo, Japan), except for the cultures of *E. coli* and *S. aureus*, which were incubated at 37 °C for 1 day under aerobic conditions. For the bioassay, bacterial suspensions containing 1 × 10^5^ colony-forming units (CFU) mL^−1^ were prepared in EG agar (Eiken Chemical, Tokyo) using the 24 h subcultures in BHI broth. The cell density was estimated by measuring the turbidity.

**Table 1:** Minimum inhibition concentration of *C. tora* seed-derived solvent fractions against harmful intestinal bacteria using a microtiter plate-based antibacterial assay (µg mL^−1^)

Harmful intestinal bacterial strains							
Fraction	***E. coli*** ATCC 11775	***C. perfringens*** ATCC 13124	***S. aureus*** ATCC 12600	***B. fragilis*** ATCC 25285	***C. difficile*** ATCC 9689	***S. typhimurium*** ATCC 13311	***C. paraputrificum*** ATCC 25780
Methanol ext	5.70 ± 0.50	2.37 ± 1.59	2.04 ± 1.00	2.69 ± 1.44	4.79 ± 0.38	2.11 ± 0.17	4.58 ± 1.40
Hexane fr	2.79 ± 0.49	2.42 ± 0.06	2.13 ± 0.60	3.32 ± 0.16	3.46 ± 0.09	2.85 ± 0.84	2.85 ± 0.56
Chloroform fr	5.82 ± 0.78	4.50 ± 1.55	5.11 ± 0.20	4.77 ± 0.14	5.13 ± 0.21	10.0 ± 0.00	10.0 ± 0.00
Ethyl acetate fr	2.40 ± 0.25	2.48 ± 0.14	4.57 ± 0.57	4.97 ± 0.36	5.18 ± 0.20	5.23 ± 0.34	5.24 ± 0.31
Butanol fr	> 20	> 20	> 20	> 20	> 20	> 20	> 20
Water fr	> 40	> 40	> 40	> 40	> 40	> 40	> 40

Values are mean ± SD (*n* = 3). Superscript letters within a column indicate statistical groupings based on Bonferroni-adjusted comparisons (*p* < 0.05). Values sharing the same letter are not significantly different. MIC values “ > 20” and “ > 40” were not included in the statistical analysis.

### Extraction and isolation

Air-dried seeds (600 g) of *C. tora* were purchased from Boeun medicinal herb shop, Kyoungdong market (Seoul, South Korea). It was pulverized and extracted with methanol (2 × 3 L) at room temperature for 1 day and filtered. The combined filtrate was concentrated under vacuum at 40 °C to yield ~ 43.83 g of a dark brownish tar. Methanol extract (20 g) was sequentially partitioned into hexane- (0.40 g), chloroform- (0.09 g), ethyl acetate- (0.46 g), butanol- (5.03 g), and water-soluble (14.02 g) portions for subsequent bioassay (Fig. S1). The organic solvent-soluble portions were concentrated to dryness by rotary evaporation at 40 °C, and the water-soluble portion was freeze-dried.

The most active hexane-soluble fraction (3 g) was chromatographed on a 70 × 5.5 cm silica gel column (300 g) and eluted with a gradient of hexane and ethyl acetate [(10:0 (1 L), 10:1 (1 L), 9:1 (1 L), 7:3 (2 L), 5:5 (1 L), 3:7 (1 L), and 1:9 (1 L) by volume] and finally with methanol (2 L) to provide 33 fractions (each about 250 mL). Column fractions were monitored by TLC on silica gel plates with hexane and ethyl acetate (8:2 by volume). Fractions with similar *R*_*f*_ values on the TLC plates were pooled. Spots were detected by spraying with 2% H_2_SO_4_ and then heating on a hot plate. Fractions 2–5 (212 mg) were pooled and purified by preparative TLC [hexane:ethyl acetate (7:3) by volume] to yield compound 1 (60 mg, *R*_*f*_ = 0.72). Fractions 6–10 (96 mg) were purified by preparative TLC [hexane:ethyl acetate (7:3) by volume] to provide compound 2 (32 mg, *R*_*f*_ = 0.65). The active fractions 11–16 (519 mg) were pooled and recrystallized in methanol at − 4 °C to afford compound 3 (23 mg, *R*_*f*_ = 0.62).

A preparative HPLC was used for the separation of the constituents from the active fractions 17–23 (321 mg) and 24–29 (227 mg). The column was a 21.2 mm × 250 mm Prodigy ODS (Phenomenex, Torrance, CA, USA) using a mobile phase of acetonitrile and water (8:2 by volume) at a flow rate of 1.5 mL min^−1^. Chromatographic separations were monitored using a UV detector at 254 nm. Finally, active principles 4 and 5 (4 and 3.7 mg) were isolated at retention times of 10.89 and 12. 85 min.

### Growth-inhibiting assay

A microtiter plate-based bioassay in sterile 96-well plates was used to evaluate the MICs of the test materials against the organisms (Casey et al. 2004). In brief, initial test materials were prepared in dimethylsulfoxide (DMSO), and twofold serial dilutions were then performed in 50 µL of EG broth. Subsequently, 10 µL of bacterial suspension of each strain was added. Ciprofloxacin (Sigma-Aldrich, St. Louis, MO, USA) served as a positive control and was similarly prepared. Negative controls consisted of 5 µL of the DMSO solution, which was verified not to affect bacterial growth in control experiments. The plates were incubated at 37 °C under anaerobic conditions in a Hirayama chamber as stated previously, except for the plates of *S. aureus* and *E. coli*, which were incubated at 37 °C under aerobic conditions. After 24 h of incubation, 10 µL of resazurin solution (270 mg resazurin in 40 mL sterile distilled water) was added to each well. For endpoint determination, resazurin dye (0.015%) was added as a redox indicator after incubation. A color change from blue to pink indicated bacterial growth, whereas the absence of color change (retained blue color) was interpreted as complete growth inhibition, corresponding to the MIC value. The MIC assay was validated using the reference strain *E. coli* ATCC 25922 as a quality control organism to confirm the accuracy and reproducibility of the microtiter plate-based method.

### Scanning electron microscopy

Both chemically untreated (control) and treated bacterial samples were centrifuged at 3000 g at 4 °C for 5 min. The bacterial pellets were primarily fixed in Karnovsky’s fixative (2% glutaraldehyde (v/v) and 2% paraformaldehyde (v/v) in 0.05 M sodium cacodylate buffer, pH 7.2) (Bednarska et al. 2016). The samples were incubated at 4 °C in darkness for 2–4 h and then washed three times with the same buffer. Second fixing was performed with 1% OsO_4_ (w/v) in the same buffer at 4 °C for 2 h. Fixed samples were washed two times with the same buffer and distilled water. The samples were then dehydrated in a graded series of ethanol increasing concentrations up to 100% for 10 min. Finally, samples were substituted with hexamethyldisilazane and dried in a Bio-Rad E3000 critical point drying machine (Cambridge, MA, USA). Drying methods can influence the preservation of surface structures; we used HMDS as a gentle drying approach. Although HMDS drying is widely applied in SEM sample preparation, it may introduce minor shrinkage or superficial artifacts. This consideration is important because accurate interpretation of membrane disruption relies on distinguishing treatment-induced damage from preparation-related effects. To ensure comparability, all treated and control samples were processed under identical conditions, minimizing preparation bias. Specimens were imaged using a JEOL JSM-5410LV scanning electron microscope (Tokyo) at 20 kV.

### Data analysis

MIC was defined as the lowest concentration that visually inhibited bacterial growth using the resazurin indicator. All experiments were performed in triplicate, and the results are presented as mean ± standard deviation (SD; *n* = 3). MIC values were statistically compared using Bonferroni-adjusted comparisons (*p* < 0.05) to determine significant differences among treatments. Superscript letters within a column indicate statistically homogeneous groups based on these comparisons (*p* < 0.05). Values sharing the same superscript letter are not significantly different from one another.

### Protein structure retrieval and preparation

Protein structures associated with antibiotic resistance were selected for molecular docking analysis as an exploratory, hypothesis-generating approach. Experimentally determined crystal structures for *E. coli* MCR-1 and blaCTX-M and *S. aureus* BlaZ were retrieved from the Protein Data Bank (PDB) (Alkhatabi et al. 2022). All remaining target proteins—*Bacteroides fragilis* CepA and NimB; *C. difficile* Erm(B) and Tet(M); *Clostridium paraputrificum* Erm(B) and Tet(M); *Clostridium perfringens* Erm(B) and Tet(M); *Salmonella typhimurium* BlaTEM and QnrB; and *S. aureus* MecA—were obtained from the AlphaFold Protein Structure Database (Jumper et al. 2021). AlphaFold structures are computational predictions rather than experimentally determined proteins; their use represents a methodological limitation. Although AlphaFold generally provides high-confidence backbone predictions, flexible regions and active-site conformations may differ from native structures, particularly for resistance proteins with dynamic catalytic pockets. Consequently, docking results derived from these models should be interpreted only as preliminary indicators of potential binding compatibility and not as evidence of target engagement or functional inhibition. All structures were energy-minimized and refined using Discovery Studio 2021. Protein identifiers (PDB or AlphaFold) are listed in Table S1.

### Ligand selection and preparation

Five anthraquinone derivatives, rhein, aloe-emodin, physcion, emodin, and anthraquinone-2-carboxylic acid, were isolated from *C. tora* L., and their 3D structures were retrieved from PubChem (Kim et al. 2020). Ciprofloxacin was selected as the reference drug. Ligands were energy-minimized and converted to PDBQT format using PyRx (Dallakyan and Olson 2015).

### Docking studies

Molecular docking was performed using AutoDock Vina integrated with PyRx software (Dallakyan and Olson 2015). Blind docking was employed as an exploratory approach to allow unbiased scanning of the entire protein surface for potential binding pockets, including both known catalytic sites and possible allosteric regions. This strategy was selected because several resistance proteins examined in this study (e.g., mecA, tet(M)) possess flexible or multi-domain architectures in which secondary binding pockets may contribute to inhibitory interactions. Although active-site focused docking is appropriate for well-defined catalytic regions such as those in β-lactamases, blind docking was used here as an initial screening method to avoid constraining the search space prematurely. For each target, grid boxes were set large enough to encompass the whole protein surface. Docking scores (binding affinities in kcal mol^−1^) were used to evaluate predicted interaction strength, with more negative values indicating stronger predicted binding. Metabolites were ranked relative to ciprofloxacin as a reference ligand.

## Results

### Isolation of active principles

**Fig. 1:**
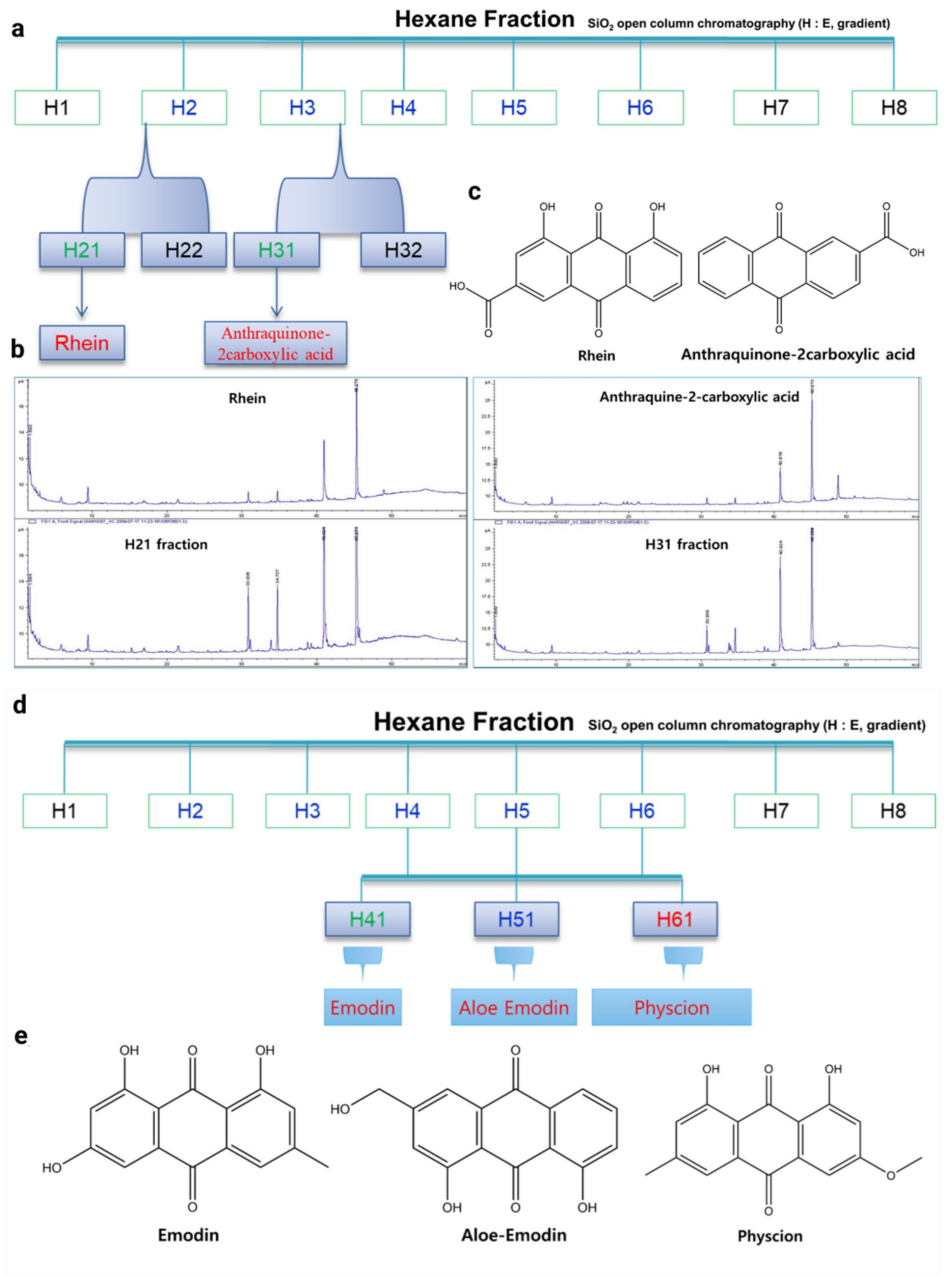
Isolation and identification of anthraquinones from *Cassia tora*. **a**, **d** The active hexane fraction was used to isolate and identify anthraquinones through silica gel chromatography. **b** HPLC chromatograms validating the identity of rhein and anthraquinone-2-carboxylic acid (ACA). **c**, **e** Chemical structures of rhein, ACA, emodin, aloe-emodin, and physcion

Paper disk diffusion bioassay-guided fractionation of *C. tora* seed extract afforded six active principles identified by spectroscopic analyses, including MS and NMR. The active principles were characterized as rhein (1), physcion (2), anthraquinone-2-carboxylic acid (3), emodin (4), and aloe-emodin (5) (Fig. 1). These metabolites were identified based on the following evidence.

Emodin (4) was identified based on the following evidence: orange needles; UV (EtOH): _max_ = 241; ^1^H NMR (DMSO, 600 MHz): δ 2.50 (1H, s), 3.33 (3H, s), 6.55 (1H, d, *J* = 2.34 Hz), 7.06 (1H, d, *J* = 2.34 Hz), 7.11 (1H, s), 7.42 (1H, s), 11.96 (1H, s), 12.03 (1H, s); ^13^C NMR (DMSO, 150 MHz): δ 21.4 q, 107.8 d, 108.7 d, 108.8 s, 113.2 s, 120.4 d, 124.0 d, 132.7 s, 135.0 s, 148.1 d, 161.3 d, 164.4 d, 165.5 s, 181.2 s, 189.6 s, EI-MS (70 eV), *m*/*z* (rel. int.): 354 [M]^+^ (100, base peak), 323, 203, 178, 161, 149, 135, 122. Rhein (1): orange needles; UV (EtOH): _max_ = 254; ^1^H NMR (DMSO, 600 MHz): δ 2.53 (1H, s), 3.33 (1H, s), 7.37 (1H, d, *J* = 8.28 Hz), 7.71 (1H, m), 7.79 (1H, d* J* = 7.92 Hz), 8.00 (1H, s), 11.86 (1H, s) 13.81 (1H, s); ^13^C NMR (DMSO, 150 MHz): δ 116.2 s, 118.6 s, 118.7 d, 119.4 d, 124.1 d, 124.5 d, 133.2 s, 133.7 s, 137.5 d, 138.2 s, 161.1 d, 161.4 d; 165.4 s, 180.9 s, 191.3 d; EI-MS (70 eV), *m*/*z* (rel. int.): 208 [M]^+^ (100, base peak), 193, 177, 165, 134, 77, 69. Physcion (2): orange needles; UV (EtOH): _max_ = 220; ^1^H NMR (DMSO, 600 MHz): δ 2.43 (3H, s), 3.94 (3H, s), 6.89 (1H, d,* J* = 2.10 Hz), 7.21 (2H, s), 7.54 (1H, s), 12.01 (1H, s), 12.13 (1H, s); ^13^C NMR (DMSO, 150 MHz): δ 21.5 d, 56.4 d, 106.6 d, 107.6 t, 109.4 d, 109.8 s, 113.4 s, 120.5 t, 124.2 t, 132.8 s, 134.8 s, 148.5 s, 161.4 d, 164.4 d, 181.2 s, 190.0 s; EI-MS (70 eV), *m*/*z* (rel. int.): 223 [M]^+^ (100, base peak), 208, 180, 167, 152, 113, 96, 72.

### Extracts against harmful intestinal bacteria

Table 1 presents the MICs of *C. tora* seed-derived solvent fractions against selected harmful intestinal bacterial strains, determined using a microtiter plate-based antibacterial assay. Among all tested fractions, the hexane fraction exhibited the strongest antibacterial activity, with MIC values ranging from 2.13 ± 0.60 to 3.46 ± 0.09 µg mL^−1^, followed closely by the ethyl acetate fraction (2.40 ± 0.25 to 5.24 ± 0.31 µg mL^−1^). The methanol extract and chloroform fraction showed moderate inhibition (2.04 ± 1.00 to 10.0 ± 0.0 µg mL^−1^), while the butanol and water fractions demonstrated negligible antibacterial activity (MIC > 20 and > 40 µg mL^−1^, respectively). Significance ranking (Bonferroni-adjusted, *p* < 0.05) indicated that the hexane and ethyl acetate fractions differed significantly from less active fractions. These results suggest that non-polar to moderately polar fractions are enriched with bioactive metabolites responsible for the pronounced antibacterial effects of *C. tora* seeds.

### Extracts against probiotic strains

Table 2 summarizes the MICs of *C. tora* seed-derived solvent fractions against probiotic strains, determined by a microtiter plate-based assay. Overall, the chloroform and ethyl acetate fractions demonstrated moderate inhibitory effects, with MIC values ranging from 2.5 ± 1.25 to 7.5 ± 2.41 µg mL^−1^, particularly against *Bifidobacterium bifidum*, *Bifidobacterium longum*, and *Bifidobacterium infantis*. The hexane fraction showed relatively mild inhibition (7.5–10.0 µg mL^−1^), while the methanol extract exhibited uniformly weak activity across all tested strains (MIC = 10.0 µg mL^−1^). In contrast, the butanol and water fractions showed no observable inhibitory activity (MIC > 20 and > 40 µg mL^−1^, respectively). Bonferroni-adjusted significance (*p* < 0.05) indicated that the chloroform and ethyl acetate fractions differed significantly from the less active methanol, butanol, and water fractions. These findings suggest that *C. tora* solvent fractions exert only moderate antibacterial effects against probiotic strains, maintaining a degree of relatively moderate activity toward pathogenic species.

**Table 2:** Minimum inhibition concentration of *C. tora* seed-derived solvent fractions against probiotic strains using a microtiter plate-based antibacterial assay (µg mL^−1^)

Fraction	Probiotic strains						
	***C. butyricum*** ATCC 25779	***B. bifidum*** ATCC 29521	***L. acidophilus*** ATCC 4356	***L. casei*** ATCC 393	***B. longum*** ATCC 15707	***B. infantis*** ATCC 25962	***B. breve*** ATCC 15700
**Methanol ext**	10.0 ± 0.0	10.0 ± 0.0	10.0 ± 0.0	10.0 ± 0.0	10.0 ± 0.0	10.0 ± 0.0	10.0 ± 0.0
**Hexane fr**	9.20 ± 1.17	7.50 ± 2.76	10.0 ± 0.0	10.0 ± 0.0	10.0 ± 0.0	7.50 ± 2.73	10.0 ± 0.0
**Chloroform fr**	10.0 ± 0.0	2.54 ± 1.25	10.0 ± 0.0	5.22 ± 3.12	2.50 ± 2.04	5.22 ± 3.10	5.15 ± 2.01
**Ethyl acetate fr**	4.67 ± 0.84	4.91 ± 2.20	5.43 ± 1.32	5.76 ± 2.41	5.21 ± 1.84	7.52 ± 1.41	5.31 ± 1.75
**Butanol fr**	> 20	> 20	> 20	> 20	> 20	> 20	> 20
**Water fr**	> 40	> 40	> 40	> 40	> 40	> 40	> 40

Values are mean ± SD (*n* = 3). Superscript letters within a column indicate statistical groupings based on Bonferroni-adjusted comparisons (*p* < 0.05). Values sharing the same letter are not significantly different. MIC values “ > 20” and “ > 40” were not included in the statistical analysis.

### Active principles against harmful intestinal bacteria

**Fig. 2:**
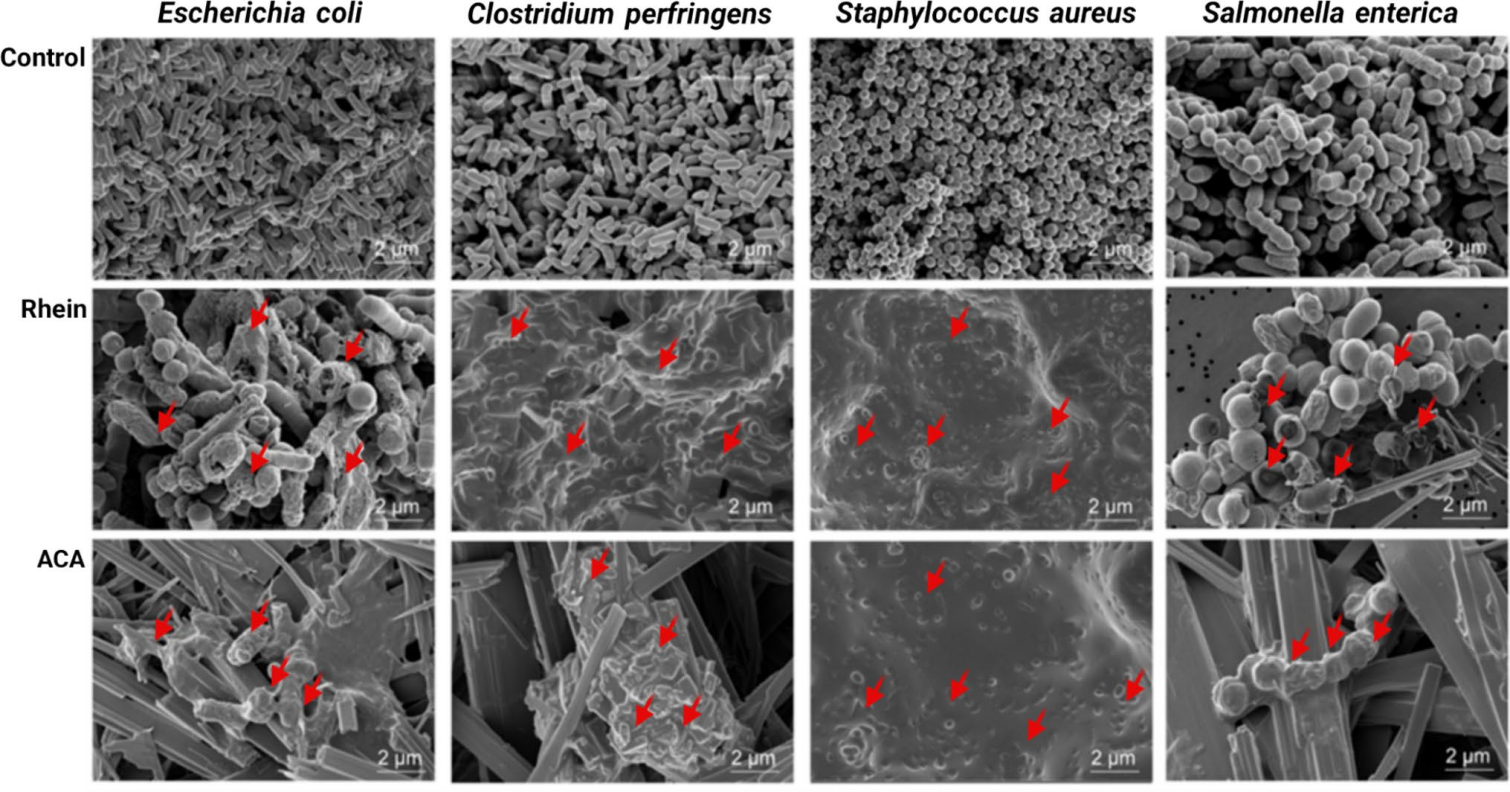
SEM analysis of bactericidal effects. Scanning electron micrographs of *E. coli*, *C. perfringens*, *S. aureus*, and *S. enterica*. Panels show untreated controls compared with cells treated with rhein or ACA. Red arrows indicate significant membrane damage

Among the *C. tora* seed-derived active principles tested, anthraquinone-2-carboxylic acid and rhein demonstrated the most potent antibacterial activity across all bacterial strains (Table 3). Anthraquinone-2-carboxylic acid showed the strongest and broadest inhibition, with MIC values ranging from 0.47 ± 0.01 to 0.77 ± 0.01 µg mL^−1^, being highly effective against *E. coli*, *C. perfringens*, *B. fragilis*, and *C. difficile* (Fig. 2). Rhein displayed comparable potency (0.47–0.96 µg mL^−1^), particularly against *S. typhimurium* and *C. paraputrificum*. Emodin demonstrated moderate activity, showing its greatest effect against *C. perfringens* (0.53 µg mL^−1^) and reduced efficacy toward *B. fragilis* and *S. typhimurium* (2.12 µg mL^−1^). In contrast, aloe-emodin and physcion exhibited relatively weaker but broad-spectrum inhibition, with MIC values between 1.3 and 3.0 µg mL^−1^. The standard antibiotic ciprofloxacin showed the lowest MICs (0.06–0.27 µg mL^−1^) and served as a positive control. Statistical analysis (Bonferroni-adjusted, *p* < 0.05) confirmed significant differences among the tested metabolites, establishing the overall potency order as anthraquinone-2-carboxylic acid > rhein > emodin > aloe-emodin ≈ physcion. Collectively, these findings indicate that *C. tora* anthraquinones, especially anthraquinone-2-carboxylic acid and rhein, possess strong and selective antibacterial effects against both Gram-positive and Gram-negative intestinal pathogens.

**Table 3:** Minimum inhibition concentration of *C. tora* seed-derived active metabolites toward harmful intestinal bacteria using microtiter plate-based antibacterial assay (µg mL^−1^)

Harmful intestinal bacterial strains							
Fraction	***E. coli*** ATCC 11775	***C. perfringens*** ATCC 13124	***S. aureus*** ATCC 12600	***B. fragilis*** ATCC 25285	***C. difficile*** ATCC 9689	***S. typhimurium*** ATCC 13311	***C. paraputrificum*** ATCC 25780
Anthraquinone-2-carboxylic acid	0.47 ± 0.01	0.47 ± 0.01	0.961 ± 0.02	0.47 ± 0.01	0.47 ± 0.01	0.47 ± 0.01	0.77 ± 0.01
Rhein	0.96 ± 0.05	0.96 ± 0.05	0.963 ± 0.05	0.96 ± 0.05	0.96 ± 0.05	0.47 ± 0.02	0.70 ± 0.04
Emodin	1.06 ± 0.06	0.53 ± 0.03	1.063 ± 0.06	2.12 ± 0.11	1.06 ± 0.06	2.12 ± 0.11	1.59 ± 0.08
Aloe-emodin	2.96 ± 0.10	2.96 ± 0.10	2.468 ± 0.08	2.96 ± 0.10	2.46 ± 0.08	1.48 ± 0.05	1.97 ± 0.06
Physcion	1.33 ± 0.10	2.66 ± 0.20	2.217 ± 0.17	2.66 ± 0.20	1.77 ± 0.13	1.77 ± 0.13	1.33 ± 0.10
Ciprofloxacin	0.07 ± 0.003	0.12 ± 0.00	0.062 ± 0.00	0.15 ± 0.006	0.27 ± 0.01	0.079 ± 0.00	0.20 ± 0.00

Values are mean ± SD (*n* = 3). Superscript letters within a column indicate statistical groupings based on Bonferroni-adjusted comparisons (*p* < 0.05). Values sharing the same letter are not significantly different.

### Active principles against probiotic strains of bacteria

The MICs of *C. tora* anthraquinone metabolites against probiotic strains, including *Clostridium butyricum*, *B. bifidum*, *Lactobacillus acidophilus*, *Lactobacillus casei*, *Bifidobacterium longum*, *Bifidobacterium infantis*, and *Bifidobacterium breve*, are presented in Table 4. Among the tested metabolites, anthraquinone-2-carboxylic acid demonstrated the lowest MIC values (1.45–4.15 µg mL^−1^), indicating mild inhibitory effects toward probiotic bacteria, particularly *C. butyricum* and *B. bifidum*. Rhein exhibited moderate inhibition (3.70–7.77 µg mL^−1^), with slightly higher sensitivity observed in *B. infantis* and *B. bifidum*. Emodin showed variable activity, with MICs between 4.68 and 6.63 µg mL^−1^, while aloe-emodin and physcion displayed comparatively weak inhibition (6.51–14.45 µg mL^−1^), suggesting minimal effects on beneficial gut bacteria. The reference antibiotic ciprofloxacin displayed the strongest inhibition (0.02–0.08 µg mL^−1^), serving as a positive control. Statistical analysis using Bonferroni-adjusted significance (*p* < 0.05) confirmed significant differences among metabolites, with anthraquinone-2-carboxylic acid showing consistently lower MICs compared to other anthraquinones. These findings indicate that *C. tora* anthraquinones exhibit only moderate activity against the probiotic strains, suggesting a preferential inhibition of pathogenic species over beneficial gut microbiota.

**Table 4:** Minimum inhibitory concentration of *C. tora* seed-derived metabolites against probiotic strains using a microtiter plate-based antibacterial assay (µg mL^−1^)

Fraction	Probiotic strains						
	***C. butyricum*** **ATCC 25779**	***B. bifidum*** **ATCC 29521**	***L. acidophilus*** **ATCC 4356**	***L. casei*** **ATCC 393**	***B. longum*** **ATCC 15707**	***B. infantis*** **ATCC 25962**	***B. breve* ATCC 15700**
**Anthraquinone-2-carboxylic acid**	1.55 ± 0.03	1.45 ± 0.13	1.79 ± 0.12	4.15 ± 0.24	3.85 ± 0.48	2.53 ± 0.12	2.82 ± 0.20
**Rhein**	5.17 ± 0.29	3.78 ± 0.18	7.77 ± 0.13	6.20 ± 0.22	7.14 ± 0.23	3.70 ± 0.14	5.41 ± 0.40
**Emodin**	6.37 ± 0.35	6.63 ± 0.25	4.68 ± 0.21	5.64 ± 0.16	6.50 ± 0.45	6.11 ± 0.05	5.51 ± 0.08
**Aloe-emodin**	6.91 ± 0.23	8.35 ± 0.24	8.96 ± 0.49	7.22 ± 0.54	6.78 ± 0.37	6.51 ± 0.25	6.65 ± 0.46
**Physcion**	9.31 ± 0.72	12.49 ± 0.88	14.45 ± 1.04	9.91 ± 0.29	10.36 ± 0.55	10.99 ± 0.21	9.90 ± 0.65
**Ciprofloxacin**	0.02 ± 0.00	0.03 ± 0.00	0.032 ± 0.00	0.05 ± 0.00	0.06 ± 0.00	0.08 ± 0.00	0.05 ± 0.00

Values are mean ± SD (*n* = 3). Superscript letters within a column indicate statistical groupings based on Bonferroni-adjusted comparisons (*p* < 0.05). Values sharing the same letter are not significantly different.

### Selectivity index analysis reveals microbiota-sparing antibacterial activity of anthraquinones

The selectivity index (SI) analysis revealed that anthraquinone-2-carboxylic acid and other anthraquinone derivatives exhibited selective antibacterial activity against pathogenic bacteria while largely sparing probiotic strains (Table 5). Anthraquinone-2-carboxylic acid showed moderate yet consistent selectivity, with SI values of 3.09–8.83 against *E. coli* and 1.51–4.32 against *S. aureus*, depending on the probiotic strain evaluated. Notably, higher selectivity was observed against *S. typhimurium*, where SI values increased to 7.87–15.19 in the presence of *B. infantis* and *B. longum*. Among the tested compounds, rhein demonstrated the highest overall SI values, ranging from 8.09 to 16.53 against *S. typhimurium* and 10.20 to 15.19 in *B. longum*-associated conditions. Emodin also displayed strong pathogen-specific activity, particularly against *C. perfringens* (10.40–12.51) and *C. difficile* (up to 6.25). In contrast, aloe-emodin consistently showed lower SI values, generally remaining below 4.0, indicating limited selectivity. Physcion exhibited moderate but stable activity with SI values ranging from 7.00 to 10.86 against *E. coli* and 7.44 to 9.39 against *C. paraputrificum.* Conversely, the reference antibiotic ciprofloxacin showed uniformly low SI values (0.07–1.29) across all tested strains, reflecting non-selective antibacterial activity. Overall, these findings identify rhein, emodin, and anthraquinone-2-carboxylic acid as promising microbiota-friendly antimicrobial candidates, with superior SI profiles compared to ciprofloxacin.

**Table 5:** Selectivity index (SI) of anthraquinone derivatives and ciprofloxacin against harmful bacterial strains in the presence of probiotic bacterial strains

Harmful strain	Probiotic strain	SI					
		**Anthraquinone-2-carboxylic acid**	**Rhein**	**Emodin**	**Aloe-emodin**	**Physcion**	**Ciprofloxacin**
***E. coli***	***C. butyricum***	3.30	5.39	6.01	2.33	7.00	0.29
	***B. bifidum***	3.09	3.94	6.25	2.82	9.39	0.43
	***L. acidophilus***	3.81	8.09	4.42	3.03	10.86	0.46
	***L. casei***	8.83	6.46	5.32	2.44	7.45	0.71
	***B. longum***	8.19	7.44	6.13	2.29	7.79	0.86
	***B. infantis***	5.38	3.85	5.76	2.20	8.26	1.14
	***B. breve***	6.00	5.64	5.20	2.25	7.44	0.71
***C. perfringens***	***C. butyricum***	3.30	5.39	12.02	2.33	3.50	0.17
	***B. bifidum***	3.09	3.94	12.51	2.82	4.70	0.25
	***L. acidophilus***	3.81	8.09	8.83	3.03	5.43	0.27
	***L. casei***	8.83	6.46	10.64	2.44	3.73	0.42
	***B. longum***	8.19	7.44	12.26	2.29	3.89	0.50
	***B. infantis***	5.38	3.85	11.53	2.20	4.13	0.67
	***B. breve***	6.00	5.64	10.40	2.25	3.72	0.42
***S. aureus***	***C. butyricum***	1.61	5.37	5.99	2.80	4.20	0.32
	***B. bifidum***	1.51	3.93	6.24	3.38	5.63	0.48
	***L. acidophilus***	1.86	8.07	4.40	3.63	6.52	0.52
	***L. casei***	4.32	6.44	5.31	2.93	4.47	0.81
	***B. longum***	4.01	7.41	6.11	2.75	4.67	0.97
	***B. infantis***	2.63	3.84	5.75	2.64	4.96	1.29
	***B. breve***	2.93	5.62	5.18	2.69	4.47	0.81
***B. fragilis***	***C. butyricum***	3.30	5.39	3.00	2.33	3.50	0.13
	***B. bifidum***	3.09	3.94	3.13	2.82	4.70	0.20
	***L. acidophilus***	3.81	8.09	2.21	3.03	5.43	0.21
	***L. casei***	8.83	6.46	2.66	2.44	3.73	0.33
	***B. longum***	8.19	7.44	3.07	2.29	3.89	0.40
	***B. infantis***	5.38	3.85	2.88	2.20	4.13	0.53
	***B. breve***	6.00	5.64	2.60	2.25	3.72	0.33
***C. difficile***	***C. butyricum***	3.30	5.39	6.01	2.81	5.26	0.07
	***B. bifidum***	3.09	3.94	6.25	3.39	7.06	0.11
	***L. acidophilus***	3.81	8.09	4.42	3.64	8.16	0.12
	***L. casei***	8.83	6.46	5.32	2.93	5.60	0.19
	***B. longum***	8.19	7.44	6.13	2.76	5.85	0.22
	***B. infantis***	5.38	3.85	5.76	2.65	6.21	0.30
	***B. breve***	6.00	5.64	5.20	2.70	5.59	0.19
***S. typhimurium***	***C. butyricum***	3.30	11.00	3.00	4.67	5.26	0.25
	***B. bifidum***	3.09	8.04	3.13	5.64	7.06	0.38
	***L. acidophilus***	3.81	16.53	2.21	6.05	8.16	0.41
	***L. casei***	8.83	13.19	2.66	4.88	5.60	0.63
	***B. longum***	8.19	15.19	3.07	4.58	5.85	0.76
	***B. infantis***	5.38	7.87	2.88	4.40	6.21	1.01
	***B. breve***	6.00	11.51	2.60	4.49	5.59	0.63
***C. paraputrificum***	***C. butyricum***	2.01	7.39	4.01	3.51	7.00	0.10
	***B. bifidum***	1.88	5.40	4.17	4.24	9.39	0.15
	***L. acidophilus***	2.32	11.10	2.94	4.55	10.86	0.16
	***L. casei***	5.39	8.86	3.55	3.66	7.45	0.25
	***B. longum***	5.00	10.20	4.09	3.44	7.79	0.30
	***B. infantis***	3.29	5.29	3.84	3.30	8.26	0.40
	***B. breve***	3.66	7.73	3.47	3.38	7.44	0.25

### Comparative analysis of binding affinity

Molecular docking was employed as a preliminary in silico approach to explore potential interactions between anthraquinone derivatives from C. *tora* L. and selected bacterial resistance-associated proteins (Table 6). The predicted binding free energies ranged from − 6.3 to − 9.3 kcal mol^−1^, indicating variable binding propensities across ligands and targets. Across nearly all protein targets, rhein, physcion, and emodin consistently produced lower (more favorable) binding energies than ciprofloxacin. For example, against *B. fragilis* cepA β-lactamase, rhein (− 8.5 kcal mol^−1^), physcion (− 8.4 kcal mol^−1^), and emodin (− 8.3 kcal mol^−1^) outperformed ciprofloxacin (− 6.8 kcal mol^−1^). A similar pattern was evident for *E. coli* blaCTX-M, *S. aureus* blaZ, and mecA, as well as *Clostridium* spp. tet(M) targets, where most anthraquinone derivatives achieved binding affinities 1–2 kcal mol^−1^ stronger than the control drug.

**Table 6:** Predicted binding affinities (kcal mol^−1^) of anthraquinone derivatives from *C. tora* L. and ciprofloxacin against antibiotic-resistance proteins

Name	Protein	Binding affinity (kcal mol^−1^)					
		**Rhein**	**Aloe-emodin**	**Physcion**	**Emodin**	**Anthraquinone-2-carboxylic acid**	**Ciprofloxacin (control)**
*B. fragilis*	cepA	− 8.5	− 8	− 8.4	− 8.3	− 8.3	− 6.8
	nimB	− 7.4	− 7.1	− 7.5	− 7.5	− 7.7	− 6.5
*C. difficile*	erm(B)	− 7.8	− 7.5	− 7.3	− 7.2	− 7.5	− 6.7
	tet(M)	− 7.4	− 7.1	− 7.2	− 7.1	− 6.8	− 6.4
*C. paraputrificum*	erm(B)	− 7	− 7	− 7.2	− 7	− 8.1	− 6.4
	tet(M)	− 8.2	− 7.8	− 8.2	− 8.2	− 8.2	− 8
*C. perfringens*	erm(B)	− 6.3	− 6.3	− 6.5	− 6.7	− 6.6	− 6.2
	tet(M)	− 7.9	− 7.5	− 7.8	− 7.6	− 8.4	− 6.8
*E. coli*	mcr-1	− 7.1	− 6.5	− 6.8	− 6.9	− 7.2	− 6.5
	blaCTX-M	− 8	− 7.8	− 7.8	− 7.9	− 7.6	− 7.1
*S. typhimurium*	blaTEM	− 9	− 7.6	− 9.2	− 9.3	− 8	− 6.7
	qnrB	− 7.1	− 6.8	− 7	− 7	− 6.9	− 6.9
*S. aureus*	blaZ	− 8	− 7.8	− 8	− 7.9	− 7.9	− 7.6
	mecA	− 7.4	− 7.2	− 7.7	− 7.6	− 8.5	− 7.5

The most pronounced effects were observed for β-lactamase determinants. Emodin bound *S. typhimurium* blaTEM with the highest affinity recorded (− 9.3 kcal mol^−1^), followed closely by physcion (− 9.2 kcal mol^−1^) and rhein (− 9.0 kcal mol^−1^), whereas ciprofloxacin bound with only − 6.7 kcal mol^−1^. Anthraquinone-2-carboxylic acid displayed the strongest binding to *C. perfringens* tet(M) (− 8.4 kcal mol^−1^) and *S. aureus* mecA (− 8.5 kcal mol^−1^), further highlighting its potential role against tetracycline and methicillin resistance mechanisms. By contrast, relatively weaker interactions were predicted with erm(B) of *C. perfringens* (− 6.3 to − 6.7 kcal mol^−1^ across ligands) and qnrB of *S. typhimurium* (− 6.8 to − 7.1 kcal mol^−1^), where ligand affinities were only marginally superior to ciprofloxacin. Likewise, mecA of *S. aureus* showed the lowest binding with rhein and aloe-emodin, comparatively, suggesting some target selectivity among the anthraquinones. We further validated the docking results by performing a comparative analysis with the native ligand at the same active site of the blaZ β-lactamase protein from *S. aureus* (PDB ID: 1ALQ). Structural analysis of the blaZ protein revealed that the catalytic pocket is predominantly composed of key residues such as SER176 and LYS177 (Fig. S2a). The native ligand exhibited a docking score of − 2.3 kcal mol^−1^ and interacted with these essential residues, serving as a reference point for evaluating the binding affinity of the selected phytocompounds (Fig. S2b). Among all tested metabolites, rhein demonstrated the strongest binding affinity with a docking score of − 4.3 kcal mol^−1^, forming stable hydrogen bond interactions with SER176, LYS177, and ASN161 (Fig. S2c). Aloe-emodin and anthraquinone-2-carboxylic acid also showed favorable docking scores of − 3.9 and − 3.7 kcal mol^−1^, respectively, interacting with residues including LYS175, LYS177, and SER176 (Fig. S2d, g). Emodin and physcion displayed moderate binding affinities of − 3.5 and − 3.1 kcal mol^−1^, maintaining hydrogen bonding and hydrophobic interactions within the active site (Fig. S2e, f). In contrast, the conventional antibiotic ciprofloxacin exhibited a docking score of − 2.4 kcal mol^−1^, which was similar to the native ligand but lower than most metabolites tested (Fig. S2h). These results suggest that anthraquinone-based phytochemicals, particularly rhein, aloe-emodin, and anthraquinone-2-carboxylic acid, possess stronger binding affinities toward the blaZ β-lactamase protein compared with the standard drug, indicating their potential as alternative β-lactamase inhibitors.

Importantly, these docking results reflect predicted binding interactions only and do not demonstrate functional inhibition of the targeted resistance proteins. Moreover, several targets were modeled using AlphaFold-predicted structures and explored using blind docking, which are suitable for exploratory hypothesis generation but may not accurately represent active-site conformations or binding dynamics. Therefore, the numerical docking scores should not be interpreted as definitive evidence of target engagement or antibacterial mechanism.

## Discussion

The gastrointestinal tract, a known reservoir for resistant genes, facilitates the transfer of resistance mechanisms to pathogenic bacteria. To address this crisis, natural products from medicinal plants have emerged as valuable sources of novel antimicrobial agents. *C. tora* has been traditionally used in Chinese and Ayurvedic medicine for various health benefits, including improving vision and acting as a cardiotonic, hypolipidemic, aperient, antiasthmatic, and diuretic agent. The seeds of *C. tora*, a widely utilized medicinal plant, are rich in bioactive metabolites known for their antimicrobial, antioxidant, and anti-inflammatory properties (Awasthi et al. 2015). Despite its established traditional use in treating gastrointestinal disorders and infections, the plant’s bioactive components, particularly anthraquinones, remain underexplored. A deeper understanding of the molecular interactions between these bioactive metabolites and bacterial targets is crucial for developing effective treatment strategies (Farha and Brown 2016).

Recent studies have highlighted the antibacterial potential of lactic acid bacteria (LAB) isolated from traditional fermented foods, which show strong inhibition against *E. coli* and *Salmonella* spp. (Vasudha and Devaraja 2023). Similar antimicrobial trends were observed in North Karnataka fermented foods, where LAB isolates demonstrated broad-spectrum inhibition against *E. coli*, *S. aureus*, *Listeria monocytogenes*, and *Salmonella typhi* (Chakra et al. 2024; 2025). Additionally, LAB derived from fermented milk exhibit functional probiotic activities, such as β-galactosidase production, that support gut function and inhibit enteric pathogens (Chakra PS, 2024). These findings support the relevance of exploring natural antimicrobial systems, including anthraquinones from *C. tora*. This is the first comparative study to isolate and evaluate individual anthraquinones from *C. tora* seeds for their selective antibacterial activity against pathogenic intestinal bacteria. Unlike previous reports that tested crude plant extracts, this work integrates bioassay-guided fractionation with molecular docking validation, demonstrating that anthraquinone-2-carboxylic acid and rhein exhibit stronger affinity toward bacterial resistance proteins than ciprofloxacin. Specifically, bioassay-guided fractionation was employed to isolate and identify six anthraquinones: rhein, anthraquinone-2-carboxylic acid, emodin, aloe-emodin, and physcion, whose structures were confirmed using NMR analysis. These anthraquinones exhibit a diverse range of bioactive properties, contributing to their antimicrobial, anticancer, and anti-inflammatory potential. Rhein demonstrates potent antibacterial activity, with the ability to disrupt bacterial membrane integrity, making it effective against antibiotic-resistant strains, while also offering anti-inflammatory and hepatoprotective benefits. Anthraquinone-2-carboxylic acid is pH-responsive and capable of inhibiting biofilm formation, positioning it as a promising therapeutic for gastrointestinal infections with minimal impact on beneficial microbiota. Emodin and aloe-emodin have shown notable efficacy in inhibiting bacterial quorum sensing and biofilm formation, which helps prevent persistent infections (Peng et al. 2022). Their hepatoprotective and anticancer properties further enhance their therapeutic versatility, particularly in inflammatory bowel conditions. Physcion stands out for its strong antifungal and antibacterial effects and potential applications in dermatology as well as a chemopreventive agent in cancer treatment (Luo et al. 2024). Collectively, these metabolites present significant potential in managing multidrug-resistant infections through mechanisms such as quorum-sensing inhibition, bacterial membrane disruption, and antioxidant effects.

In our results, anthraquinone-2-carboxylic acid demonstrated the strongest antibacterial activity among the tested metabolites, with very low MICs against pathogenic strains (0.47–0.96 µg mL^−1^). This compound also inhibited probiotic bacteria at higher concentrations (1.45–4.15 µg mL^−1^), indicating a pattern of *relative selectivity* rather than absolute specificity. The calculated selectivity index (SI = MIC_probiotic/MIC_pathogen) ranged from approximately 3 to 10, suggesting a favorable but not complete preferential inhibition of harmful pathogens. This dose-dependent selectivity likely arises from the functional groups present in anthraquinones, such as hydroxyl and carboxyl moieties, which might enhance interactions with bacterial membranes (Dell’Annunziata et al., 2022).

Solvent polarity also influenced the extraction of bioactive compounds. Non-polar fractions (hexane and chloroform) exhibited notable activity, including moderate inhibition of *Bifidobacterium longum* and *Lactobacillus acidophilus*, likely due to the extraction of hydrophobic metabolites such as anthraquinones and flavonoids, which are known to disrupt membrane integrity. Methanol, hexane, and ethyl acetate fractions showed moderate to high antibacterial activity, reflecting their efficiency in extracting metabolites with antimicrobial potential. In contrast, polar fractions such as butanol and water exhibited minimal activity, suggesting limited extraction of active metabolites. Overall, although the metabolites show stronger activity toward pathogens, their measurable effects on probiotic strains emphasize the need for dose optimization and validate the use of selectivity indices to interpret therapeutic potential.

The observed membrane disruption (SEM) and predicted protein binding (docking) suggest multiple potential targets; however, definitive mechanistic studies are required to establish the primary mode of action.

Interestingly, our findings compared with previous studies on *C. tora* leaf extracts showed significant differences in antibacterial activity and bioactive potential. A previous study reported that *C. tora* leaf methanolic extract exhibited antibacterial activity against *E. coli* (25.20 ± 0.49 mm inhibition zone) but was less effective against *S. aureus* and *P. aeruginosa* (Ogunjobi and Moses 2013). In contrast, the seed-derived anthraquinones in our study demonstrated superior efficacy, with much lower MIC values, highlighting the higher potency of the seed extracts in targeting intestinal pathogens. These differences in activity can be attributed to the distinct phytochemical compositions of the seeds and leaves. Seeds are known to contain a higher concentration of anthraquinones and flavonoids, which have potent antibacterial mechanisms, whereas leaves contain secondary metabolites with strong antioxidant properties, beneficial for enhancing overall health rather than exerting targeted antimicrobial effects. Furthermore, the previous study demonstrated that supplementing *C. tora* leaf extract in poultry drinking water significantly improved growth performance by reducing lipid peroxidation and enhancing antioxidant enzyme activity (Sahu et al. 2017). While the leaf extracts contributed to poultry health, their limited antibacterial spectrum suggests a different mode of action, primarily focusing on antioxidant-driven health benefits rather than direct bacterial inhibition. On the other hand, our findings on *C. tora* seed-derived anthraquinones suggest their potential as targeted antimicrobial agents for human therapeutic applications, offering a more selective approach to combating antibiotic-resistant intestinal pathogens while maintaining gut microbiota balance (Sahu et al. 2017). These findings emphasize the need for targeted selection of plant parts based on their specific bioactive properties to maximize therapeutic potential. Further research exploring the synergistic effects of combining seed and leaf extracts could unlock novel strategies for addressing AMR and enhancing gut health. *C. tora* seed extracts have also been used for the green synthesis of AgNPs, which showed notable antibacterial activity against *S. aureus* (Saravanakumar et al. 2015). However, the anthraquinones isolated in our study exhibited markedly stronger activity at much lower concentrations, indicating that the seed phytochemicals themselves may provide more potent antimicrobial effects than AgNP formulations.

Molecular docking is widely applied as an exploratory computational approach to predict potential ligand–protein interactions and to support hypothesis generation (Dhandapani et al., 2024). Although more negative binding affinity values are often associated with increased complex stability (Samad et al., 2022), such predictions do not imply functional inhibition or biological relevance. In the present study, C. *tora* anthraquinones displayed favorable predicted binding energies (− 6.3 to − 9.3 kcal mol^−1^) toward several resistance-associated proteins, including β-lactamases (blaTEM, blaCTX-M) and tetracycline/methicillin resistance determinants (tet(M), mecA). However, these observations are based on static docking models, including AlphaFold-predicted protein structures and blind docking protocols, which may not accurately capture active-site conformations, protein flexibility, or catalytic dynamics. Consequently, the numerical docking scores should be interpreted only as preliminary indicators of possible binding compatibility, not as evidence of target engagement or mechanism. These computational results therefore serve solely to prioritize candidate protein–ligand pairs for future validation, which would require focused docking, molecular dynamics simulations, and direct biochemical or enzymatic assays.

In addition, compared to synthetic antibiotics like Ciprofloxacin, *C. tora* anthraquinones offer distinct advantages, including selective activity and multifaceted mechanisms of action. While Ciprofloxacin exhibited superior MIC values in this study, its broad-spectrum activity poses risks of disrupting beneficial microbiota, a limitation not observed with *C. tora* metabolites. Combination therapies involving *C. tora* anthraquinones and conventional antibiotics could potentially reduce the dosage of synthetic drugs required and lower resistance development risks. These comparisons further underscore the versatility of *C. tora* seeds as a valuable resource for developing diverse antibacterial strategies, ranging from phytochemical-based therapies to nanomaterial applications (Nawabjohn et al. 2022). Their selective activity against harmful pathogens and ability to spare beneficial gut microbiota position them as promising candidates for addressing AMR. To advance their clinical application, further research is needed to evaluate their pharmacokinetics, bioavailability, and toxicity in animal models. In addition to the in silico docking analysis, experimental validation is essential. The interaction with multidrug-resistant target proteins should be confirmed through comprehensive in vitro assays, followed by in vivo studies to substantiate the biological efficacy and therapeutic relevance of the metabolites. Additionally, exploring their synergistic potential with existing antibiotics could pave the way for combination therapies that minimize resistance development.

## Conclusion

This study demonstrates the potent antibacterial activity of bioactive anthraquinones isolated from *C. tora* seeds against a range of pathogenic intestinal microorganisms. Among the tested metabolites, anthraquinone-2-carboxylic acid and rhein exhibited the highest antibacterial potency, with MIC values as low as 0.46 µg mL^−1^, supporting their potential as alternative therapeutic agents for managing antibiotic-resistant intestinal infections. While these compounds were more active against pathogenic strains, they also displayed measurable inhibitory effects on probiotic bacteria at higher concentrations. The findings, therefore, indicate a relative or modest, rather than complete, absence of activity toward beneficial gut flora. This differential susceptibility is reflected in favorable SI values, suggesting that therapeutic windows can be optimized to preserve microbiome balance. Molecular docking analyses provided preliminary hypotheses consistent with the in vitro findings, indicating that *C. tora* anthraquinones may exhibit stronger predicted binding affinities than ciprofloxacin toward key bacterial resistance proteins (e.g., blaTEM, blaCTX-M, tet(M), and mecA). These computational predictions suggest potential molecular targets but do not confirm functional inhibition. Importantly, validation through dedicated in vitro enzymatic assays, such as β-lactamase inhibition assays, is required to determine whether these metabolites can effectively interfere with resistance proteins in biological systems.

Overall, the anthraquinones from *C. tora* show strong antibacterial activity against pathogens and moderate effects on probiotic bacteria, emphasizing the importance of balancing antimicrobial efficacy with microbiome safety in therapeutic design. The present study is limited to in vitro and in silico evaluations; therefore, in vivo validation, pharmacokinetic assessment, and toxicity profiling are essential to confirm translational applicability. Future studies must evaluate mammalian cell cytotoxicity to determine a true therapeutic index, investigate potential synergistic interactions with conventional antibiotics, optimize formulation strategies for improved bioavailability, and integrate extended molecular dynamics simulations to prioritize *C. tora* for clinical development.

## Supplementary Information

Below is the link to the electronic supplementary material.

ESM 1 (DOCX 718 KB)

## Data Availability

All data generated or analyzed during this study are included in this published article [and its supplementary information files].

## References

[CR1] Alkhatabi HA, Zohny SF, Shait Mohammed MR, Choudhry H, Rehan M, Ahmad A, Ahmed F, Khan MI (2022) Venetoclax-resistant MV4-11 leukemic cells activate PI3K/AKT pathway for metabolic reprogramming and redox adaptation for survival. Antioxidants (Basel, Switzerland) 11. 10.3390/antiox11030461 10.3390/antiox11030461 PMC8944541 35326111

[CR2] Anthony WE, Burnham CD, Dantas G, Kwon JH (2021) The gut microbiome as a reservoir for antimicrobial resistance. J Infect Dis 223:S209-s213 33326581 10.1093/infdis/jiaa497 PMC8206794

[CR3] Awasthi VK, Mahdi F, Chander R, Khanna AK, Saxena JK, Singh R, Mahdi AA, Singh RK (2015) Hypolipidemic activity of *Cassia tora* seeds in hyperlipidemic rats. Indian J Clin Biochem 30:78–83 25646045 10.1007/s12291-013-0412-2 PMC4310838

[CR4] Bednarska NG, van Eldere J, Gallardo R, Ganesan A, Ramakers M, Vogel I, Baatsen P, Staes A, Goethals M, Hammarström P, Nilsson KP, Gevaert K, Schymkowitz J, Rousseau F (2016) Protein aggregation as an antibiotic design strategy. Mol Microbiol 99:849–865 26559925 10.1111/mmi.13269

[CR5] Burbure VS, Baheti AM, Deshmukh CD, Wani MS, Deshpande M (2020) Phytochemical and pharmacological profile of Cassia tora. J Hosp Pharm 15(4):72

[CR6] Casey JT, O’Cleirigh C, Walsh PK, O’Shea DG (2004) Development of a robust microtiter plate-based assay method for assessment of bioactivity. J Microbiol Methods 58:327–334 15279937 10.1016/j.mimet.2004.04.017

[CR7] Chakra PS, Yalagondanahalli CN, Gayathri D (2025) Antibacterial and enzymatic potential of lactic acid bacteria from traditional fermented foods. J Health Allied Sci NU 15:352–359. 10.25259/JHS-2024-7-12-R3-(1478)

[CR8] Chakra Siddappa P, Devaraja G (2024) Isolation, diversity and antibacterial efficacy of potential indigenous lactic acid bacteria from North Karnataka, India. Biomedicine 44(2):228–235

[CR9] Dallakyan S, Olson AJ (2015) Small-molecule library screening by docking with PyRx. Methods Mol Biol 1263:243–250 25618350 10.1007/978-1-4939-2269-7_19

[CR10] Dell’Annunziata F, Folliero V, Palma F, Crudele V, Finamore E, Sanna G, Manzin A, De Filippis A, Galdiero M, Franci G (2022) Anthraquinone Rhein Exhibits Antibacterial Activity against Staphylococcus aureus. Appl Sci 12:8691. 10.3390/app12178691

[CR11] Dhandapani S, Samad A, Liu Y, Wang R, Balusamy SR, Perumalsamy H, Kim YJ (2024) Coprisin/compound K conjugated gold nanoparticles induced cell death through apoptosis and ferroptosis pathway in adenocarcinoma gastric cells. ACS Omega 9(24):25932–25944. 10.1021/acsomega.4c00554 10.1021/acsomega.4c00554 PMC11190908 38911731

[CR12] Farha MA, Brown ED (2016) Strategies for target identification of antimicrobial natural products. Nat Prod Rep 33:668–680 26806527 10.1039/c5np00127g

[CR13] Islam MT, Ali ES, Uddin SJ, Shaw S, Islam MA, Ahmed MI, Shill MC, Karmakar UK, Yarla NS, Khan IN, Billah MM, Pieczynska MD, Zengin G, Malainer C, Nicoletti F, Gulei D, Berindan-Neagoe I, Apostolov A, Banach M, Yeung AWK, Atanasov AG (2018) Phytol: a review of biomedical activities. Food Chem Toxicol 121:82–94. 10.1016/j.fct.2018.08.032 10.1016/j.fct.2018.08.032 30130593

[CR14] Jesudason T (2024) WHO publishes updated list of bacterial priority pathogens. Lancet Microbe 5:100940 39079540 10.1016/j.lanmic.2024.07.003

[CR15] Jumper J, Evans R, Pritzel A, Green T, Figurnov M, Ronneberger O, Tunyasuvunakool K, Bates R, Žídek A, Potapenko A, Bridgland A, Meyer C, Kohl SAA, Ballard AJ, Cowie A, Romera-Paredes B, Nikolov S, Jain R, Adler J, Back T, Petersen S, Reiman D, Clancy E, Zielinski M, Steinegger M, Pacholska M, Berghammer T, Bodenstein S, Silver D, Vinyals O, Senior AW, Kavukcuoglu K, Kohli P, Hassabis D (2021) Highly accurate protein structure prediction with AlphaFold. Nature 596:583–589 34265844 10.1038/s41586-021-03819-2 PMC8371605

[CR16] Kim S, Covington A, Pamer EG (2017) The intestinal microbiota: antibiotics, colonization resistance, and enteric pathogens. Immunol Rev 279:90–105 28856737 10.1111/imr.12563 PMC6026851

[CR17] Kim S, Chen J, Cheng T, Gindulyte A, He J, He S, Li Q, Shoemaker BA, Thiessen PA, Yu B, Zaslavsky L, Zhang J, Bolton EE (2020) PubChem in 2021: new data content and improved web interfaces. Nucleic Acids Res 49:D1388–D1395 10.1093/nar/gkaa971 PMC7778930 33151290

[CR18] Krishnaprasad VH, Kumar S (2024) Antimicrobial resistance: an ultimate challenge for 21st century scientists, healthcare professionals, and policymakers to save future generations. Naunyn Schmiedebergs Arch Pharmacol 67:15927–15930. 10.1021/acs.jmedchem.4c02002 10.1021/acs.jmedchem.4c02002 39238216

[CR19] Luo H, Ji X, Zhang M, Ren Y, Tan R, Jiang H, Wu X (2024) Aloe-emodin: progress in pharmacological activity, safety, and pharmaceutical formulation applications. Mini Rev Med Chem 24:1784–1798 38639277 10.2174/0113895575298364240409064833

[CR20] Nawabjohn MS, Sivaprakasam P, Anandasadagopan SK, Begum AA, Pandurangan AK (2022) Green synthesis and characterisation of silver nanoparticles using *Cassia tora* seed extract and investigation of antibacterial potential. Appl Biochem Biotechnol 194:464–478 34611854 10.1007/s12010-021-03651-4

[CR21] Ogunjobi A, Moses A (2013) Antimicrobial activity of *Senna alata* and *Phyllanthus amarus*. Drug Dev Res 7:198–202

[CR22] Peng F, Fang F, Xiang R, Liu D (2022) Engineering properties of *Cassia tora* L. seeds and meal as a function of moisture content. Sci Rep 12:8651 35606481 10.1038/s41598-022-12748-7 PMC9126907

[CR23] Sahu J, Koley KM, Sahu BD (2017) Attribution of antibacterial and antioxidant activity of *Cassia tora* extract toward its growth promoting effect in broiler birds. Vet World 10:221–226 28344406 10.14202/vetworld.2017.221-226 PMC5352848

[CR24] Samad A, Huq MA, Rahman MS (2022) Bioinformatics approaches identified dasatinib and bortezomib inhibit the activity of MCM7 protein as a potential treatment against human cancer. Sci Rep 12(1):1539. 10.1038/s41598-022-05621-0 10.1038/s41598-022-05621-0 PMC8795118 35087187

[CR25] Saravanakumar A, Ganesh M, Jayaprakash J, Jang HT (2015) Biosynthesis of silver nanoparticles using *Cassia tora* leaf extract and its antioxidant and antibacterial activities. J Ind Eng Chem 28:277–281

[CR26] Seukep AJ, Tamambang FM, Matieta VY, Mbuntcha HG, Bomba FDT, Kuete V, Ndip LMA (2025) Potential of methanol extracts of Nephelium lappaceum (Sapindaceae) and Hyphaene thebaica (Arecaceae) as adjuvants to enhance the efficacy of antibiotics against critical class priority bacteria. PLoS One 20(2):e0314958. 10.1371/journal.pone.0314958 10.1371/journal.pone.0314958 PMC11819497 39937773

[CR27] Sorbara MT, Dubin K, Littmann ER, Moody TU, Fontana E, Seok R, Leiner IM, Taur Y, Peled JU, van den Brink MRM, Litvak Y, Bäumler AJ, Chaubard JL, Pickard AJ, Cross JR, Pamer EG (2019) Inhibiting antibiotic-resistant Enterobacteriaceae by microbiota-mediated intracellular acidification. J Exp Med 216:84–98 30563917 10.1084/jem.20181639 PMC6314524

[CR28] Tang S, Stasiewicz MJ, Wiedmann M, Boor KJ, Bergholz TM (2013) Efficacy of different antimicrobials on inhibition of *Listeria monocytogenes* growth in laboratory medium and on cold-smoked salmon. Int J Food Microbiol 165:265–275 23803569 10.1016/j.ijfoodmicro.2013.05.018

[CR29] Vasudha M, Devaraja g (2023) Metabolism and functional heterogeneity of fermented milk origin lactic acid bacteria for lactose intolerance. J Microbiol Biotechnol Food Sci 13:e9654

[CR30] Weiss GA, Hennet T (2017) Mechanisms and consequences of intestinal dysbiosis. Cell Mol Life Sci 74(16):2959–2977. 10.1007/s00018-017-2509-x 10.1007/s00018-017-2509-x PMC11107543 28352996

