# Supplementary material for: Antibacterial activity of bioactive anthraquinones isolated from Cassia tora L. against pathogenic intestinal microorganisms
Source: Naunyn Schmiedebergs Arch Pharmacol. 2026 Feb 13;399(7):10931–45. doi: 10.1007/s00210-026-05084-4 (PMC13152883; doi:10.1007/s00210-026-05084-4)
Supplement: Supplementary file 1 — (DOCX 718 KB) [file 210_2026_5084_MOESM1_ESM.docx]

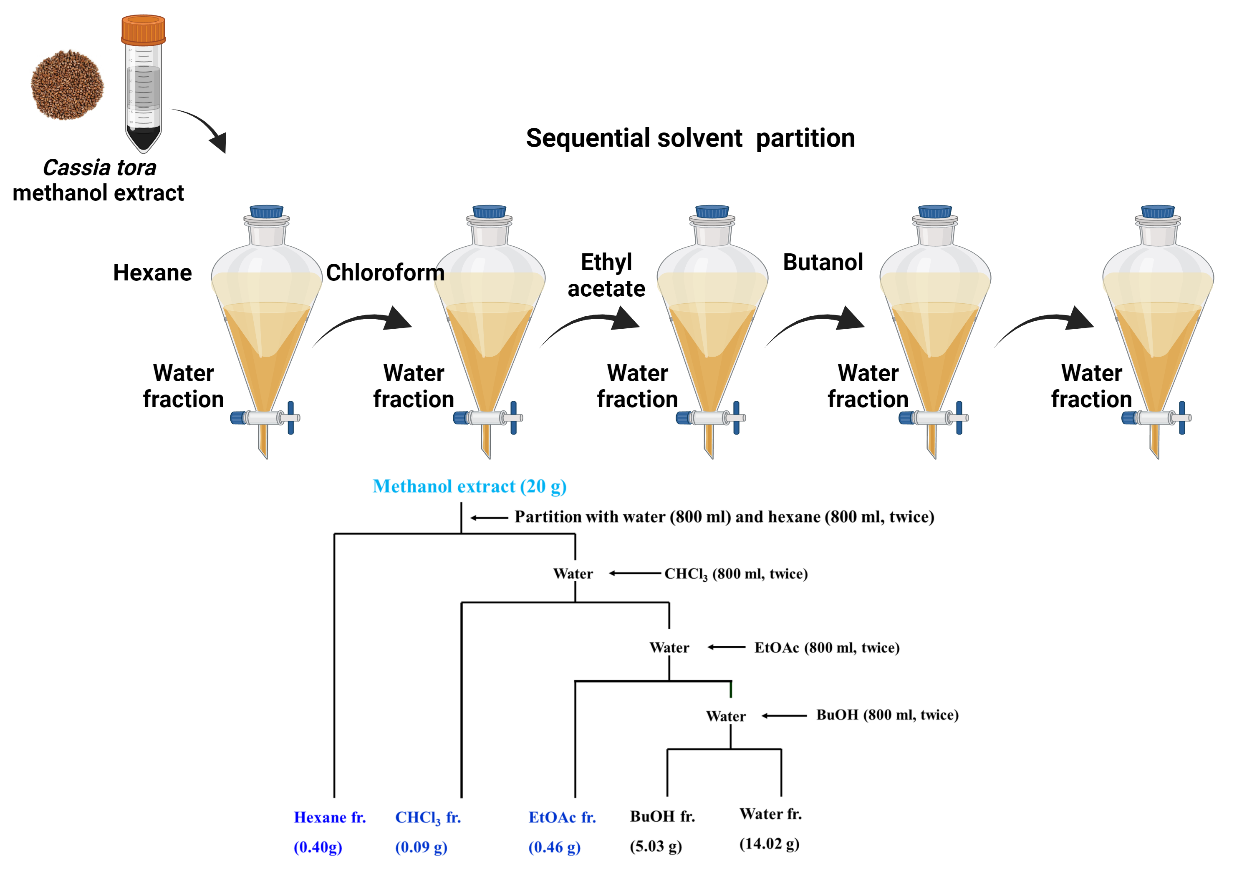


**Figure S1. Sequential solvent partition**. *C. tora* seed methanol extract was sequentially partitioned with different solvents including hexane, chloroform, ethyl acetate, butanol, based on polarity and density.


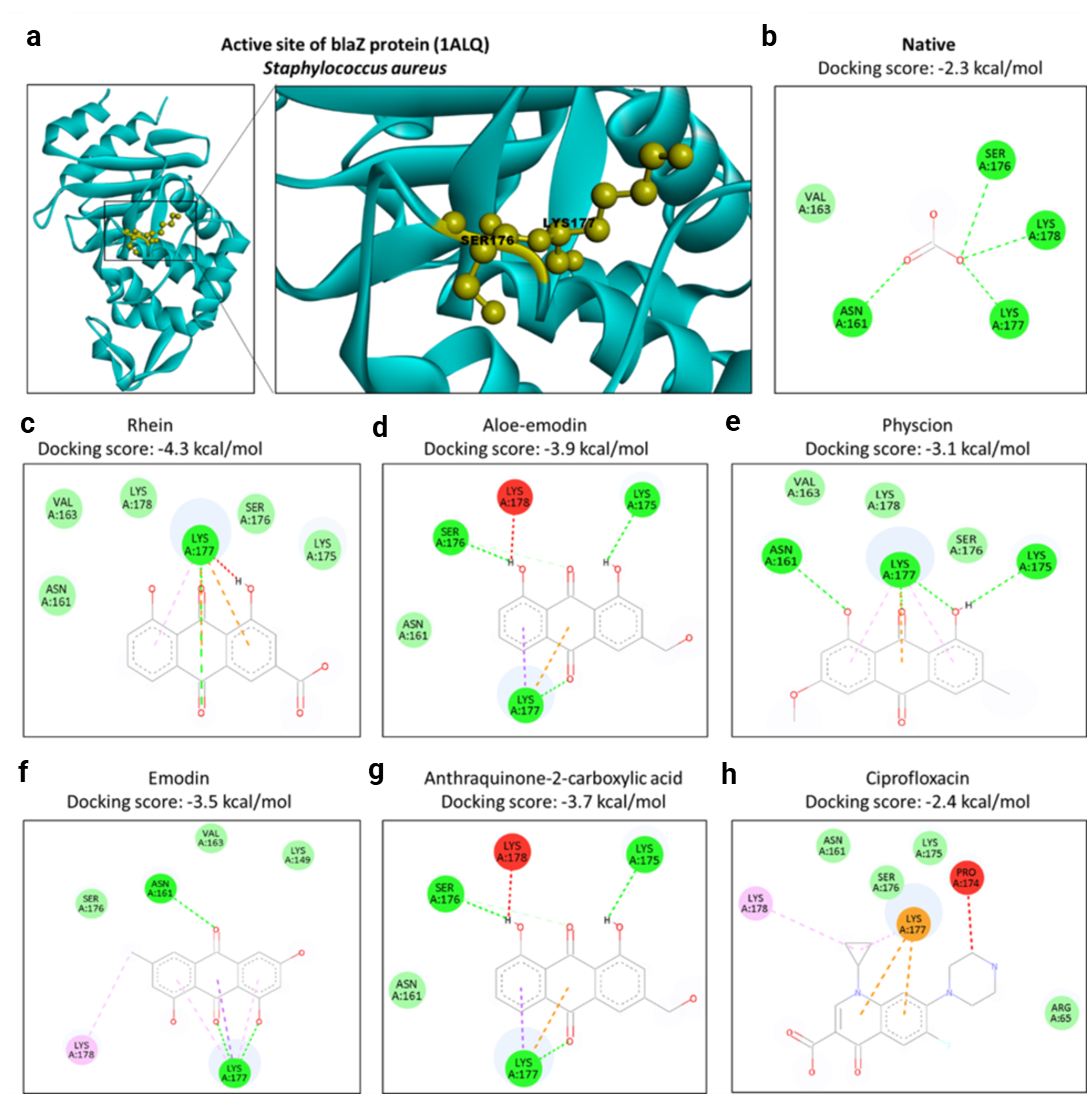


**Figure S2. Molecular docking of metabolites with the active site of blaZ β-lactamase protein from *S. aureus* (PDB ID: 1ALQ).** (a) Three-dimensional structure of blaZ protein showing the active site residues. (b) Docking pose of the native ligand exhibiting a docking score of –2.3 kcal/mol, serving as the reference standard. (c–h) Predicted binding interactions of selected phytochemicals with blaZ protein: (c) Rhein (–4.3 kcal/mol), (d) Aloe-emodin (–3.9 kcal/mol), (e) Physcion (–3.1 kcal/mol), (f) Emodin (–3.5 kcal/mol), (g) Anthraquinone-2-carboxylic acid (–3.7 kcal/mol), and (h) Ciprofloxacin (–2.4 kcal/mol, positive control). Each panel illustrates hydrogen bonds, hydrophobic interactions, and amino acid residues involved in ligand stabilization within the active pocket. Metabolites Rhein, Aloe-emodin, and Anthraquinone-2-carboxylic acid demonstrated the strongest binding affinities compared to the native ligand and ciprofloxacin.

**Table S1**. Bacterial proteins selected for molecular docking analysis, with corresponding PDB or AlphaFold structure identifiers.

| **Bacteria name** | **Protein** | **PDB/AlhphaFold identifier** |
| --- | --- | --- |
| *B. fragilis* | cepA | AF-Q57150 |
|  | nimB | AF-Q45146 |
| *C. difficile* | erm(B) | AF-Q181C1 |
|  | tet(M) | AF-K0C2H0 |
| *C. paraputrificum* | erm(B) | AF-A0A174GNF4 |
|  | tet(M) | AF-A0A6N2YJU1 |
| *C. perfringens* | erm(B) | AF-P0A4D6 |
|  | tet(M) | AF-Q9AC90 |
| *E. coli* | mcr-1 | 7YJS |
|  | blaCTX-M | 8SJ3 |
| *S. typhimurium* | blaTEM | AF-Q5QJI7 |
|  | qnrB | AF-C4NZV9 |
| *S. aureus* | blaZ | 1ALQ |
|  | mecA | AF-Q5HH86 |
